# Improving Accuracy of ACE-III Marking in the Wyre Forest Older Adult Community Mental Health Team

**DOI:** 10.1192/bjo.2025.10407

**Published:** 2025-06-20

**Authors:** Amy Moltu, Lei-Lei Tun, Dhanjeev Marrie, Tandrila Sinha

**Affiliations:** Herefordshire & Worcestershire Health & Care NHS Trust, Worcester, United Kingdom

## Abstract

**Aims:** Accurate implementation and marking of the ACE-III (Addenbrooke’s Cognitive Examination) within memory assessments is vital in informing clinical diagnoses. The 100-mark cognitive test is subject to various nuances in its marking criteria that can easily be overlooked or misinterpreted.

Given noticeable discrepancy in staff completion, we aimed to review the accuracy of scoring of the ACE-IIIs completed by the team. We sought to identify the domains with the greatest variability in scoring, hypothesising that this would be the visuospatial domain, and feed this back as a teaching session with view to improving future accuracy in completion.

**Methods:** 50 patients were identified from the Wyre Forest Older Adult Community Mental Health Team who had recently (last six months) undergone an ACE-III examination, split equally between the geographic East and West of the region (as covered by different consultant psychiatrists).

Independent reviewers reviewed each ACE-III against the 2017 scoring guide. Any ambiguous cases were decided by consensus. Certain aspects of the ACE-III cannot be objectively verified retrospectively and require contemporaneous observation, therefore these questions were excluded from the analysis.

**Results:** 45 of the 50 patients had a valid ACE-III available for review. Only 44% (20/45) assessments had no identifiable marking errors.

As hypothesised, the highest failure rates occurred in the visuospatial domains, with incorrect marking in the “copying a cube” in 20% (9/45) and “clock drawing” in 18% (8/45).

Moderate failure rates were observed within the language section with “sentence writing”, being inaccurately marked in 9% (4/45) and the visuospatial task of the “infinity diagram”, with issues in 7% (3/45).

Several of the attention, memory and fluency-specific tasks saw a mistake in at least one patient.

The mistakes were generally evenly spread across patients with no single ACE-III examination accounting for a disproportionate number of errors.

**Conclusion:** Uniformity in application and marking of the ACE-III requires revision with the team in order to achieve consistency. Even marginal inaccuracies in scoring could result in an under- or over-estimation of cognitive ability and influence clinicians’ interpretation and subsequent diagnosis.

In the teaching session, the team reflected on the results and their experiences, and collectively decided on further improvement measures:

1) in-house simulation ACE-III completion with a volunteer administrator, the team lead as patient, and remaining team observing,

2) to include a bi-annual team re-training with the ACE-III training video as a refresher, and

3) for re-evaluation in approximately six months’ time to monitor improvement.

